# Volatile Profiling and Transcriptome Sequencing Provide Insights into the Biosynthesis of α-Pinene and β-Pinene in *Liquidambar formosana* Hance Leaves

**DOI:** 10.3390/genes14010163

**Published:** 2023-01-06

**Authors:** Yongquan Li, Yanfang Gao, Lin Deng, Huiming Lian, Wei Guo, Wei Wu, Bine Xue, Baobin Li, Yuzhen Su, Hui Zhang

**Affiliations:** 1 Department of Scarce and Quality Economic Forest Engineering Technology Research Center (2022GCZX002), College of Horticulture and Landscape Architecture, Zhongkai University of Agriculture and Engineering, Guangzhou 510408, China; 2 Bijie Institute of Forestry Science, Bijie 551700, China; 3 Guangdong Academy of Forestry, Guangzhou 510520, China; 4 Dongjiang Forest Farm, Heyuan 517000, China; 5 Guangdong Lingnanyuan Exploration and Design Co., Ltd., Guangzhou 510500, China

**Keywords:** *Liquidambar formosana* Hance, transcriptome, leaf fragrance, terpenoids, α-pinene, β-pinene

## Abstract

*Liquidambar formosana* Hance is a pinene-rich deciduous plant species in the Altingiaceae family that is used as a medicinal plant in China. However, the regulatory mechanisms underlying α-pinene and β-pinene biosynthesis in *L. formosana* leaves remain unknown. Here, a joint analysis of the volatile compounds and transcriptomes of *L. formosana* leaves was performed to comprehensively explore the terpene synthase (TPS) that may participate in α-pinene and β-pinene biosynthesis. Headspace solid-phase microextraction (HS-SPME) and gas chromatography–mass spectrometry (GC–MS) jointly detected volatile *L. formosana* leaves. Trees with high and low levels of both α-pinene and β-pinene were defined as the H group and L group, respectively. RNA sequencing data revealed that DXR (1-deoxy-D-xylulose-5-phosphate reductoisomerase), HDS [(E)-4-hydroxy-3-methylbut-2-eny-l-diphosphate synthase], and TPS may be the major regulators of monoterpenoid biosynthesis. We identified three TPSs (*LfTPS1, LfTPS2*, and *LfTPS3*), which are highly homologous to α-pinene and β-pinene synthases of other species in phylogenetic analysis. Four TPS genes (*LfTPS1*, *LfTPS2*, *LfTPS4*, *LfTPS5*) may be critically involved in the biosynthesis and regulation of α-pinene and β-pinene in *L. formosana*. Bioinformatic and transcriptomic results were verified using quantitative real-time PCR. We identified *LfTPS1, LfTPS2* as candidate genes for α-pinene and β-pinene biosynthesis that significantly improve the yield of beneficial terpenoids.

## 1. Introduction

*Liquidambar formosana* is a deciduous tree species in the Altingiaceae family distributed in subtropical China [1]. It is a common forest tree species widely used for forestry and landscaping [2,3]. This species produces high-quality timber and is a well-known source of pharmaceuticals and cosmetics. Diverse bioactive products (antibacterial [4,5], fruit preservation [6], anti-inflammatory [7], antioxidant [8], and antiseptic [9]) extracted from *L. formosana* leaves are widely used [10]. However, *L. formosana* is mainly used in landscaping and its utilization in other production forms is extremely deficient.

The fragrance of *L. formosana* leaves is unique. Volatile organic compounds (VOCs) from *L. formosana* account for 95.51% of the total volatile components using a solid-phase microextraction (SPME) pipeline [11]. The primary components are α-pinene, β-pinene, caryophyllene, and limonene. Diverse VOCs were identified in *L. formosana* forest varieties using thermal desorption–gas chromatography–mass spectrometry (TD–GC–MS) [12,13]; most of them were limonene, α-pinene, and β-pinene, which correlated with previous studies. Twenty-six VOCs were detected in *L. formosana* trees (including limonene, myrcene, and β-pinene) [14] using a previous method [13]. Furthermore, samples harvested in summer showed a significant decline in bacterial and fungal content [15], and limonene, myrcene, and β-pinene significantly inhibit bacteria in vitro. There are 23 VOCs in *L. formosana, Zelkova serrate*, *Acer buergerianum* and *Ginkgo biloba*; they are composed mainly of terpenes, including longifolene, α-pinene, and longicyclene [15]. Despite differences in the experimental method and sampling time, some differences were observed in the above studies. Furthermore, leaf essential oils of *L. formosana* consist of diverse terpenes, including α-pinene, β-pinene, limonene, and phellandrene, each of which has high pharmacological activity [16,17,18]. Previous studies mainly focused on evaluating the composition of VOCs released from *L. formosana* trees and their ecological service function; however, the underlying mechanism for the biosynthesis of the representative volatiles remained elusive.

Terpenoids are the most abundant and diverse secondary metabolites in plants and are extensively used in food preservation, medicinal, and cosmetic markets; thus, their biosynthesis was investigated in recent years. Success in overexpressing monoterpene synthase genes corresponding to high amounts of plant volatile substance is likely due to gene expression matching the accumulation of their products [19,20,21]. Terpenoids are key components of the leaf fragrance of *L. formosana*. There are two terpenoid biosynthetic pathways in plants: the mevalonate (MVA) pathway in the cytoplasm, endoplasmic reticulum, and peroxisome using acetyl-CoA as a precursor [22,23] and the 2-C-methyl-D-erythritol 4-phosphate (MEP) pathway, which is distributed among the plastids and uses pyruvate and 3-phosphoglyceraldehyde (GA-3P) as precursors [24,25,26]. These two pathways are not independent of each other; rather, there is a certain degree of communication [27,28,29,30]. Terpenoids are hydrocarbons and their oxygenated derivatives are polymerized using the isoprene backbone of the 5-carbon atom as the basic unit. Isopentenyl diphosphate (IPP) and its isomer dimethylallyl diphosphate (DMAPP) are the universal precursors of terpenoids. Head-to-tail condensation of IPP and DMAPP results in the formation of geranyl diphosphate (GPP, C10), farnesyl diphosphate (FPP, C15), and geranylgeranyl diphosphate (GGPP, C20) by the action of geranyl diphosphate synthase (GPS), farnesyl diphosphate synthase (FPPS), and geranylgeranyl diphosphate synthase (GGPPS), respectively [31,32,33]. It was also reported that z-FPS synthase in wild tomato glandular hairs catalyzes the synthesis of Z,Z-FPP from IPP and DMIPP and uses it as a substrate to synthesize tomato sesquiterpenes via a sesquiterpene synthase (SBS), providing new insights into higher plant terpene biosynthesis [34]. The MVA pathway synthesizes IPP and DMIPP under the catalysis of six enzymes (AACT, HMGS, HMGR, MK, PMK, MPDC) and the MEP pathway synthesizes IPP and DMIPP under the catalysis of seven enzymes (DXS, DXR, MCT, CMK, MDS, HDS, HDR). DXS is a key rate-limiting enzyme in the MEP pathway, which controls the synthesis of monoterpenes and polyterpenes [35]. Terpene synthases (TPSs) catalyze the cyclization and rearrangement of direct precursors of terpenes, including GPP, FPP, and GGPP, while undergoing enzymatic reactions (hydroxylation, glycosylation, peroxidation, and methylation) under the action of modifying enzymes to form structurally rich terpenes [36]. The structurally rich terpenoids are also derived from the diversity of TPS enzymes. Different TPSs can also catalyze the same precursor to obtain different terpenoids. The ability of a terpenoid synthase to catalyze the formation of multiple products from the same precursor is also considered a universal characteristic of the TPS enzyme family, which is attributed to the fact that carbocationic intermediates produced during the reaction can have more than one metabolic products [37,38,39]. Terpene synthases catalyze multiple enzyme reactions, although the specific mechanisms remains unclear. Recently, sequencing and transcriptome analysis have emerged as powerful tools for investigating non-model plants. A better understanding of the biosynthesis and regulation of α-pinene and β-pinene is highly desirable given their high demand. This study performed transcriptomic analysis and VOC analysis of *L. formosana* leaves to further enrich our understanding of the molecular regulation and biosynthesis of components in leaf fragrance. The results of this study may help to select high terpenoid-yielding species using molecular marker-assisted selection.

In this study, transcriptome sequencing data analysis showed that there were 1202 DEGs in group H vs. group L, including 689 upregulated and 513 downregulated genes. Two genes (*LfTPS1*, *LfTPS2,*) containing typical TPS domains were identified from differentially expressed genes, and the expression levels of the above two genes were highly correlated with the relative content of α-pinene and β-pinene, which were defined as candidate genes regulating the biosynthesis of α-pinene and β-pinene in Liquidambar formosana leaves.

## 2. Materials and Methods

### 2.1. Materials and Instruments

*L. formosana* plants were kindly provided by Dr. Li (Dongjiang Forestry Station, Guangdong Province, China), (114°44′ E, 23°38′ N). The field conditions included an average annual temperature of 20.9–21.5 °C, and the average annual rainfall was 1600–1900 mm. Afforestation occurred in May 2005, belongs to native broad-leaved seed generation test forest. Healthy *L. formosana* samples from 12 different individual plants were harvested between 14:00 and 16:00 in August 2021. Samples for headspace solid-phase microextraction gas chromatography–mass spectrometry (HS-SPME–GC–MS) were collected and stored at 15 °C before analysis. According to the results of HS-SPME–GC–MS, 6 plants with low and high pinene content were selected. The transcriptome sequencing materials were harvested and immediately frozen in liquid nitrogen. Later, the materials were stored at −80 °C for transcriptome sequencing and reverse transcription-quantitative polymerase chain reaction (RT-qPCR) analyses.

### 2.2. HS-SPME of Volatile Components from L. formosana Leaves

Samples were quickly ground into powder by pulverizer, approximately 1 g was weighed and immediately sealed in 20 mL SPME vials for further analysis. Solid phase microextraction fibers of DVB/CAR/PDMS (divinylbenzene/carboxen/poly-dimethylsiloxane, 50/30 μm) (Shanghai Amp Experimental Technology Co., Ltd., Shanghai, China) were used to extract the VOCs of *L. formosana*. The fibers were conditioned for 60 min according to the temperature recommended by the manufacturer. The SPME fiber was equilibrated for 30 min to account for the distance between the sample and the headspace. Then, the SPME fiber was heated at 98 °C for 33 min to extract volatile compounds from each sample. The fiber was quickly moved into the GC–MS for thermal desorption and analysis performed. Three technical replicates were used from each of the three biological replicates.

### 2.3. GC/MS Analysis

Samples were analyzed and identified using an Agilent 7890A Series GC system coupled with an Agilent 5975C Mass Selective Detector (Agilent Technologies, Santa Clara, CA, USA). Helium was used as the carrier gas at a flow rate of 1.0 mL/min with an HP-INNOWAX capillary column (30 m × 250 µm × 0.25 µm). The inlet temperature was 200 °C. The column temperature program of the GC was initially set at 40 °C for 3 min, heated to 100 °C at 3 °C/min, maintained for 5 min, increased to 200 °C at 20 °C/min, and maintained for 7 min. Mass spectrometry detection used an electron impact (EI) ionization system at 70 eV, the ionization source temperature was 280 °C, the quadrupole temperature was 150 °C, the full-scan acquisition mode was performed with a mass range of 33–350 (*m/z*), and the tuning file included standard tuning. Constituents were identified by comparing the mass spectra with the National Institute of Standards and Technology (NIST08, http://webbook.nist.gov/chemistry/ (accessed on 29 November 2022)) library and published data. Furthermore, the retention time (RT) of the standard substances for the representative compounds was measured according to the above experimental conditions. Relative percentages (RPs) were calculated to determine the proportion of each component.

### 2.4. RNA Extraction, cDNA Library Construction, and Sequencing

Total RNA was isolated from *L. formosana* leaves using TRIzol^®^ reagent (Invitrogen, Shanghai, China) following the manufacturer’s protocol, and genomic DNA was removed using DNase I (TaKara, Beijing, China). RNA quality was analyzed using an Agilent 2100 Bioanalyzer and quantified using an ND-2000 spectrophotometer (NanoDrop Technologies, Wilmington, DE, USA). A high-quality RNA sample (OD260/280 = 1.82, OD260/230 = 2.0, 28S:18S = 1.0. Total RNA > 10 µg) was used to build the sequencing library.

RNA-seq transcriptome libraries were created using 1 g of total RNA and the Illumina TruSeq RNA sample preparation kit (San Diego, CA, USA). Briefly, messenger RNA was isolated using polyA beads and fragmented using a fragmentation buffer. Illumina-indexed adaptors were ligated after cDNA synthesis, end repair, A-base addition, and ligation, according to the Illumina protocol. Libraries were size selected for cDNA target fragments of 200–300 bp on 2% low-range ultra-agarose, which were then PCR-amplified for 15 cycles with Phusion DNA polymerase (NEB). Paired-end libraries were sequenced using Illumina NovaSeq 6000 (150 bp × 2, Shanghai BIOZERON Co., Ltd., Shanghai, China) after quantification using a TBS-380 mini-fluorometer. The Illumina paired-end sequencing service was provided by Shanghai Ling En Biotechnology Co., Ltd., Shanghai, China.

### 2.5. Transcriptome Data Analysis and Annotation

Processing of the transcriptome sequence data was performed through a filtering process considering a Q-value of 30 using the fastqc and Trimmomatic toolkit. We first removed adaptor sequences, empty reads, and low-quality sequences. The total clean reads were aligned to the *L. formosana* reference genome (unpublished) using Hisat 2 software. The alignment results were quantified using the R language package feature Counts, and the read count for each gene was normalized to transcripts per million (TPM) [40]. The effects of gene length and sequencing depth can be eliminated using TPM, which can also directly compare gene expression differences between different samples. Differential expression analysis was conducted using DESeq2 v1.10.1 (Michael Love, 2016). Multiple testing hypothesis correction was performed to estimate relative expression levels. An adjusted *p*-value < 0.05 and |log2FC| ≥ 1 were used as the threshold parameters of significantly differentially expressed genes (DEGs).

Functional annotation was performed according to the Gene Ontology (GO) terms and the Kyoto Encyclopedia of Genes and Genomes (KEGG) to analyze the biosynthesis of terpenoid pathways and genetic pathways involved in this study. Pathway analysis was performed in KOBAS v3.0.3 (Dechao Bu, Haitao Luo, et al., 2017), to systematically explore the functions of DEGs. The main biological functions were determined and the terpenoid synthesis pathways were obtained. The coding sequence of key enzymes involved in this pathway was screened by searching for the gene name of the EC number.

### 2.6. Screening of TPS Genes Related to α-Pinene and β-Pinene

We initially selected DEGs that were directly annotated as TPSs by KEGG. Then, a local BLASTP analysis was performed for TPS sequences using previously reported related sequences from other plants. Then, phylogenetic analysis of the TPSs of *L. formosana* was performed in MEGA 11.0 using the neighbor-joining (NJ) method with *n* = 1000 bootstrap replicates. The TPS amino acid sequence was analyzed by MEME (https://meme-suite.org/meme/doc/meme.html; accessed on 18 March 2022) online analysis website, and the conserved motif was predicted. The predicted motif was further verified by the Pfam (https://pfam.xfam.org; accessed on 20 March 2022) online website. According to the differential genes containing specific domains, the information of phylogenetic tree and the prediction results of structural motifs, the genes containing TPS domains were screened. IBM SPSS (version 21.0; Armonk, NY, USA) was used to obtain Spearman’s correlation coefficients between terpene synthase and the content of α-pinene and β-pinene. Finally, a gene metabolite network map was established using Cytoscape (v3.9.1, Shannon P et al., 2003) [41] based on Spearman’s correlation coefficients.

### 2.7. Quantitative Real-Time PCR (qRT-PCR) Analysis

Quantitative real-time PCR was used to validate the reliability of RNA sequencing data as previously described [42]. The total RNA of the samples was extracted and reversed into cDNA for RT-qPCR analysis using applied biosystems 7500. The PCR procedure was pre-denaturation at 95 °C for 3 min, denaturation at 95 °C for 10 s, annealing at 60 °C for 30 s, 40 cycles. Three biological replicates per reaction, relative expression levels calculated using the 2^−ΔΔCt^ method, with actin serving as the reference gene. Measurements were obtained in triplicate from three biological replicates. All primers were designed using Primer Premier software (version 5.0, PREMIER Biosoft, San Francisco, CA, USA) for the six unigenes (Supplementary Table S1).

### 2.8. Graphical Analysis of Obtained Results

Microsoft Excel (2013) was used to draw pictures. Volcano map, heat map, histogram and bubble map were prepared using R (2009–2020), provided by RStudio (v4.0.3, Posit Software, PBC, Boston, MA, USA).

## 3. Results

### 3.1. Comparing the Contents of α-Pinene and β-Pinene in Different Single L. formosana Leaf Samples

**Figure 1:**
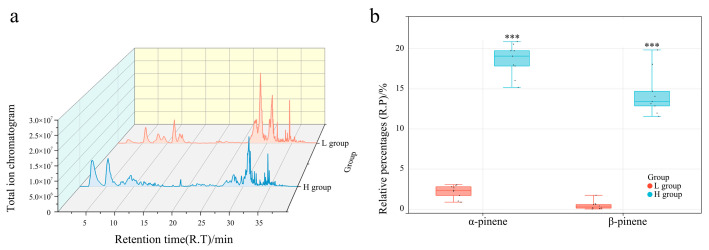
Comparison of phenotypic characteristics between L and H groups. (**a**) Total ion chromatogram of L and H group samples. (**b**) Comparison of α-pinene and β-pinene contents between L and H groups; *** *p* < 0.0001.

Volatile components in *L. formosana* leaf samples were analyzed by HS-SPME–GC–MS (Supplementary Table S2). The relative percentages (R.P.s) of each volatile organic compound were calculated using area normalization. There were individual plants with significant differences in the content of α-pinene and β-pinene (Supplementary Figure S1). Three trees with high (H) α-pinene and β-pinene contents and three trees with low (L) contents were divided into H groups (LF6 →H1; LF12 → H2; LF11 → H3) and L groups (LF2 → L1; LF7 → L2; LF5 → L3), respectively. The contents of α-pinene and β-pinene were positively correlated (Figure 1b). The contents of α-pinene and β-pinene were 1.35–2.81% and 0.24–0.87%, respectively, in the L group and 16.97–19.41% and 12.64–17.3%, respectively, in the H group. All individuals in the H group accumulated significantly higher levels of α-pinene and β-pinene.

### 3.2. Gene Expression Quantification and DEG Identification

**Figure 2:**
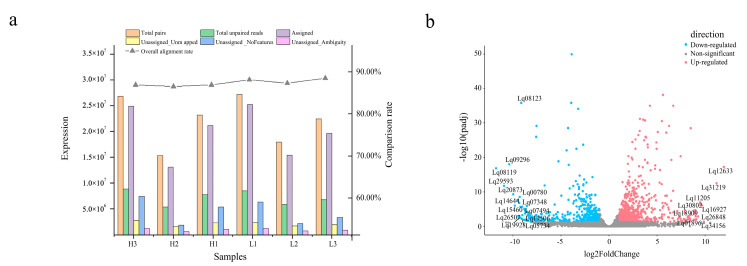
Gene expression quantification and differentially expressed gene (DEG) identification in the transcriptome. (**a**) Sequence alignment and expression quantification of sequenced samples. (**b**) Differentially expressed genes. Horizontal coordinates indicate the logarithmic values of the fold difference between H and L groups, while vertical coordinates indicate the -log_10_ value of the false discovery rate (FDR) of the difference between the two subgroups. Orange–red points and blue–green points indicate upregulated and downregulated expression in the H vs. L groups, respectively, while gray points indicate no significant differences.

The Illumina HiSeq 6000 platform was used to determine the transcriptome sequences of the six trees (H and L groups). A total of 88 Gb of raw data was obtained. Quality control filtered reads were compared to the *L. formosana* reference genome (unpublished) using Hisat 2 (Mihaela Pertea et al., 2020). The reads of unassigned unmapped, unassigned nofeatures, and unassigned ambiguity were at a lower level, and assigned reads were high enough. The overall alignment rates of H1, H2, H3, L1, L2, and L3 were 86.91%, 86.54%, 86.89%, 88.13%, 87.31%, and 88.50%, respectively (Table 1; Figure 2a). These results indicated that the transcriptome sequencing and alignment rates in this study were high enough for subsequent analyses.

The DEGs were designated according to the expression level of |log_2_(fold change)| > 1 and *p*-value < 0.05 in the H vs. L group. A total of 1202 DEGs were identified between the two groups (689 upregulated and 513 downregulated) (Figure 2b and Supplementary Table S3).

### 3.3. Gene Ontology Functional Annotation and KEGG Enrichment Analysis of DEGs

**Figure 3:**
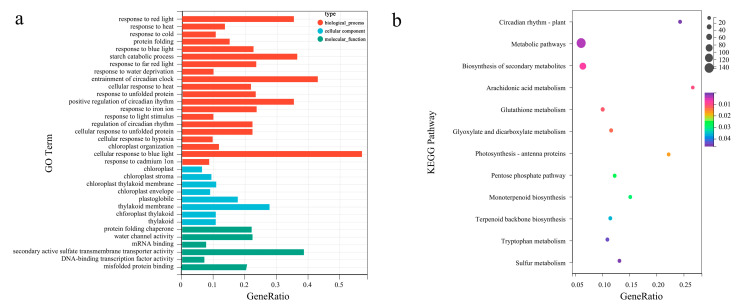
Gene Ontology (GO) functional annotation and Kyoto Encyclopedia of Genes and Genomes (KEGG) enrichment analysis of DEGs. (**a**) GO enrichment classification of differentially expressed genes. The top 10 terms with the largest number of differential genes were taken for the plot, the horizontal coordinate is the number of differential genes in that GO term, and the vertical coordinate is the specific GO term. (**b**) KEGG enrichment classification of DEGs. The horizontal coordinate is the −log10 *p*-value of the DEGs in the KEGG pathway name, and the vertical coordinate is the specific pathway name. The darker the orange–red color, the smaller the −log10 *p*-value; the darker the blue–green color, the larger the −log_10_ *p*-value.

The synthesis of related secondary metabolites may be regulated by DEGs at the transcriptional level. Gene Ontology enrichment analyses were performed to reveal the biological functions of *L. formosana* leaves to exhaustively explore the presumed function of DEGs (Supplementary Table S4). The main molecular function (MF), cellular component (CC), and biological process (BP) categories were identified. A total of 1202 DEGs were clustered into 21 biological processes, eight cellular components, and six molecular functions (Figure 3a). Most subcategories were related to the cellular response to blue light, entrainment of the circadian clock, starch catabolic processes, and positive regulation of circadian rhythm in the BP categories. The largest percentages of DEGs in the CC category were related to the thylakoid membrane, protein-folding chaperones, and plastoglobules. There were only eight subcategories enriched in cellular components. This mainly included secondary active sulfate transmembrane transporter activity and water channel activity.

Additionally, enrichment analysis focused on the metabolic pathways and the (KEGG) direct homology system [KEGG Orthology (KO)] to gain insight into the biological functions of different proteins in coordination with each other. The DEGs from the H vs. L group comparison were assigned to 13 pathways, while the top three enriched pathways were “arachidonic acid metabolism”, “circadian rhythm—plant”, and “photosynthesis—antenna proteins” (Figure 3b).

### 3.4. Identification of Genes Related to Monoterpene Biosynthesis

**Figure 4:**
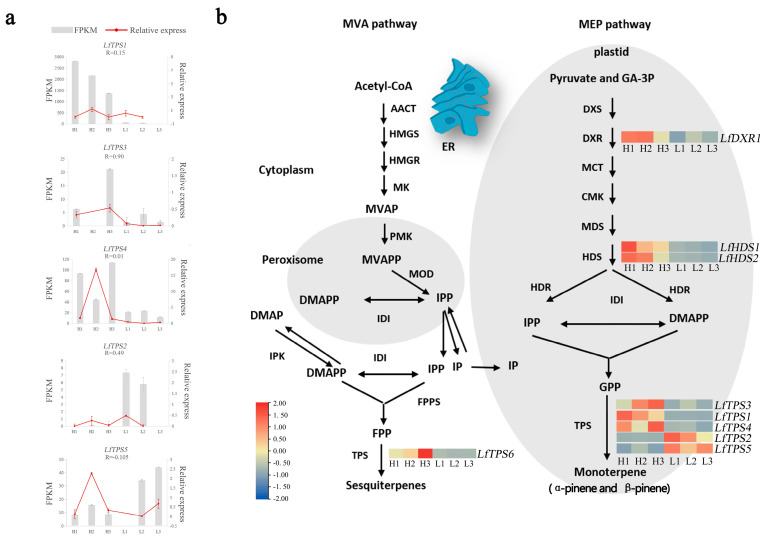
Identification of genes related to monoterpene biosynthesis. (**a**) Fragments per kilobase of exon per million reads (FPKM) and quantitative real-time polymerase chain reaction (qRT-PCR). Error bars represent the standard error (SE). “R” meaned the correlation coefficient between FPKM and relative express. (**b**) Differentially expressed genes related to terpenoid biosynthesis. Expression profiles of genes encoding enzymes involved in the terpenoid backbone biosynthesis pathway (ko00900) and monoterpenoid biosynthesis pathway (ko00902) (group H vs. group L). Enzymes related to terpenoid biosynthesis are shown in red frames. The expression pattern was shown within six columns: the 3 columns on the left represent the H group and the 3 columns on the right represent the L group. AACT, acetyl-CoA C-acetyltransferase; HMGS, hydroxymethylglutaryl-CoA synthase; HMGR, hydroxymethylglutaryl-CoA reductase; MK, mevalonate kinase; PMK, phosphomevalonate kinase; MDC, mevalonate diphosphate decarboxylase; DXS, 1-deoxy-D-xylulose-5-phosphate synthase; DXR, 1-deoxy-D-xylulose-5-phosphate reductoisomerase; MCT, 2-C-methyl-D-erythritol 4-phosphate cytidylyltransferase; CMK, 4-diphosphocytidyl-2-C-methyl-D-erythritol kinase; MDS, 2-C-methyl-D-erythritol 2,4-cyclodiphosphate synthase; HDS, (E)-4-hydroxy-3-methylbut-2-eny-l-diphosphate synthase; HDR, 4-hydroxy-3-methylbut-2-en-1-yl diphosphate reductase.

Kobas software was used to identify contigs related to the biosynthesis of the MEP and MVA pathways. KEGG metabolic pathway analysis showed that the MEP pathways related to *L. formosana* monoterpene synthesis were the terpene skeleton biosynthesis pathways (ko00900) and the monoterpene biosynthesis pathways (ko00902). We focused on the expression patterns of genes involved in terpene biosynthesis to further assess the differences in volatile terpene biosynthesis between the H and L groups (Figure 4). Nine DEGs participating in the terpene synthesis pathway showed significant activities. Gene expression of key enzymes in the MEP pathway is more active than that in the MVA pathway. These nine DEGs consisted of two homologs of HDS and one homolog of DXR. Differentially expressed genes were substantially increased in the H group compared to those in the L group. Of these, DXR (*LfDXR1*), was the highest in the H group, and the HDS (*LfHDS1, LfHDS2*), displayed the highest level in the H group. Five TPS-encoding genes related to monoterpene synthesis in the *L. formosana* transcriptome. *LfTPS1*, *LfTPS3* and *LfTPS4* were upregulated and *LfTPS2* and *LfTPS5* were downregulated in the H group. This correlated with the content of α-pinene and β-pinene. We believe that this will help identify novel genes involved in the synthesis of pathways of secondary metabolites or terpenoid biosynthesis in *L. formosana*.

Simultaneously, we identified five TPS-encoding genes related to monoterpene synthesis in the *L. formosana* transcriptome. Quantitative real-time PCR was used to detect the expression levels of five DEGs in the H and L groups (Supplementary Table S5). The trend of gene changes in qRT-PCR was roughly consistent with that detected in the transcriptome. The correlation between the expression of *LfTPS3* in each sample and qRT-QPCR reached 0.90. Three genes (*LfTPS3*, *LfTPS1*, and *LfTPS4*) exhibited similar transcriptional patterns to α-pinene and β-pinene production. This suggested that they directly affected α-pinene and β-pinene production.

**Figure 5:**
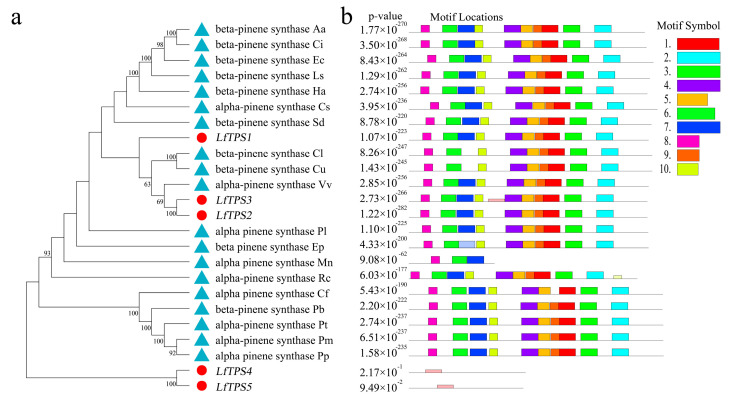

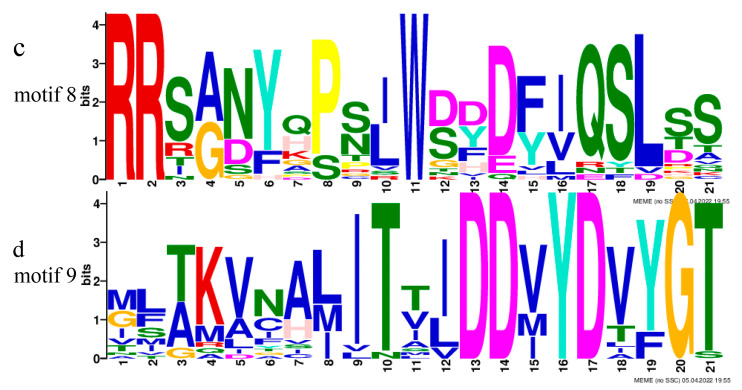
Phylogenetic analysis and correlation analysis of terpene synthases (TPSs). (**a**) Phylogenetic analysis of amino acid sequences of TPS in *L. formosana* along with other characterized TPS plants. The TPSs identified in this study are marked with red dots. (**b**) Comparison of TPS found in the *L. formosana* motif; (**c**,**d**) main motifs.

Phylogenetic analysis was performed based on published terpene synthase genes associated with the synthesis of α-pinene and β-pinene, combined with the corresponding amino acid sequences (Figure 5a). *LfTPS3*, *LfTPS2*, and *LfTPS1* were highly homologous to α-pinene and β-pinene synthases of other species, such as *Vitis vinifera*. *LfTPS4* and *LfTPS5* are members of distinct branches. Conserved motif analysis performed with the MEME program revealed that TPS members clustered in the same branch had similar motif characteristics (Figure 5b). The predicted results indicated that *LfTPS3*, *LfTPS1*, *LfTPS2*, and other published terpene synthase genes contained 10 identical motifs with the same sequence. For example, the RR (X) 8W and DD (X) 2D motifs (Figure 5c,d). An identical motif was not detected in LfTPS4 and LfTPS5. Furthermore, Pfam analysis of all motif sequences showed that motifs 1, 2, 3, 4, 5, and 9 were located in the C-terminal domain of TPS, and motifs 6, 7, and 10 were located in the N-terminal domain of TPS. *LfTPS3*, *LfTPS1*, and *LfTPS2* all contained the C-terminal and N-terminal TPS domains.

**Figure 6:**
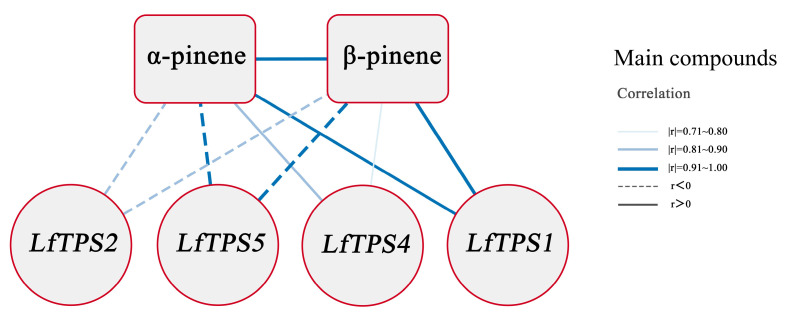
Interaction networks between the 4 TPSs of *L. formosana*, α-pinene, and β-pinene. The thicker edge line means a larger correlation coefficient, while a solid line indicates a positive correlation, and a dashed line indicates a negative correlation (using only correlation coefficients > 0.6, *p*-values < 0.05).

A correlation analysis was performed to clarify the relationship between the TPM values of the five TPS genes and terpene content and form correlation networks (Supplementary Table S6). The correlation coefficients between *LfTPS3* and α-pinene and β-pinene were >0.6, and the *p*-values were >0.05; therefore, this gene was not selected for further correlation analysis. The correlation network expression pattern showed that *LfTPS1* expression positively correlated with α-pinene and β-pinene accumulation, whereas *LfTPS5* and *LfTPS2* negatively correlated with the production of α-pinene and β-pinene. *LfTPS4* positively correlated with α-pinene production (Figure 6).

## 4. Discussion

Epigenetically related metabolites are associated with epigenetic modifications and epigenetic modification patterns vary from species to species [43]. Terpenoids are natural products that have a complex biosynthesis and release mechanism and a diverse spectrum of biological functions, which play an important role in adaptation to specific ecological conditions, information transfer, and chemical defense in plant life processes [44,45]. For example, floral volatile terpenoids attract pollinators, while toxic terpenoids are released as phytotoxins to defend against herbivores, harmful insects, pathogenic microorganisms, and other threats. Some can even mediate the interaction of plants with the surrounding biotic and abiotic factors [46,47,48,49,50]. *L. formosana* leaves are rich in terpenoids, and its primary component (pinene) has great potential for application in food preservation, and pharmaceutical and cosmetic industries. This study showed that the vast majority of volatile compounds from *L. formosana* leaves are α-pinene, β-pinene, caryophyllene, and limonene based on HS-SPME–GC–MS characterization. This correlated with previous studies [11,14,51]. In addition, the germplasm of *L. formosana* in this study contained high concentrations of α-pinene and β-pinene. This germplasm has not been effectively utilized and protected, and its leaf fragrance has not been comprehensively evaluated, hindering better exploitation and utilization of high-quality *L. formosana* resources. Pharmacological activities of α-pinene and β-pinene include antibacterial, antiviral, anti-spasmodic, anti-fungal, anti-viral, anti-cancer, anti-malarial, anti-oxidation, anti-inflammatory, and sedative effects [52,53,54].

Terpenoid content is a complex and diverse cause of species differences in various environments [55,56]. Pathways related to α-pinene and β-pinene biosynthesis and molecular regulation of *L. formosana* are not extensively elucidated compared with other well-studied plant families such as Lamiaceae [57], Pinaceae [36], and Apiaceae. High-throughput sequencing techniques have rapidly increased our knowledge and understanding of the biosynthesis and molecular regulatory frameworks of valuable phytochemicals by generating transcriptome sequence data from important plants [58]. This study performed transcriptome sequencing of six trees of *L. formosana* leaves. This provides insights into the molecular mechanisms underlying terpenoid synthesis in *L. formosana*. The H group had mostly higher expression of the DEGs relevant to the terpenoid biosynthesis pathway compared with those in the L group. This indicated that the H group had enhanced terpenoid synthesis. Terpenoids consist of IPP (C5) units that are derived from two distinguishable biochemical pathways in plants: the plastidial MEP pathway and the cytosolic acetate-MVA pathway. However, monoterpenoids are solely produced by the MEP pathway from IPP [58,59]. The MEP pathway is involved in the synthesis of IPP and DMIPP by seven enzymes (DXS, DXR, MCT, CMK, MDS, HDS, and HDR). DXS is a key enzyme in the MEP pathway and some studies suggest that DXS regulates monoterpene production and is mainly involved in the buildup of linalool, geraniol, α-terpinol, and other substances in grape aroma and *Freesia hybrida* [35,60,61]. Kiwifruit has two DXS genes, with co-expression of *DXS1* and *TPS* genes significantly increasing monoterpenoid accumulation [62]. DXS overexpression in peppermint enhances the accumulation of essential oil substances [63]. *DXR* encodes the second key enzyme in the MEP pathway, is the process’s rate-limiting enzyme, is an effective regulatory target, and is involved in terpene synthesis [64]. HDR genes influence the ultimate synthesis of terpenoids by indirectly regulating isoprene synthesis. GPPS6 is distributed in secretory gland cells and chloroplasts and indirectly regulates monoterpene biosynthesis. This study showed that the *DXR*, *HDR*, and *GPPS* genes were highly expressed in the H group, and we presumed that they are involved in the regulation of principal compounds in *L. formosana* leaf fragrance.

IPP is synthesized into monoterpenes, sesquiterpenes, and diterpenes with different carbon chain lengths by the action of isopentenyl transferases and terpene synthases. The structural diversity of terpenoids is mainly based on terpene synthases and related genes [36,65,66], and a series of monoterpenes and sesquiterpenes are synthesized by different TPSs with FPP and GPP as substrates. Phylogenetic analysis revealed that *LfTPS3*, *LfTPS2*, and *LfTPS1* are highly homologous and similar to α-pinene and β-pinene synthases of other species. Four TPS genes (*LfTPS1, LfTPS2, LfTPS4,* and *LfTPS5*) might be critically involved in the biosynthesis and regulation of α-pinene and β-pinene in *L. formosana* according to interaction networks. However, predicting the function of the TPS gene solely based on sequence similarity is rather unpromising because the sequence homology between terpene synthase proteins is independent of function [67]. Furthermore, most TPSs are multiproduct enzymes capable of producing a diverse set of compounds [68]. For example, 2-limonene synthase in spearmint (*Mentha spicata*) and *peppermint* (*Mentha* × *piperita*) produces α-pinene and (-)-β-pinene, together with the monocyclic product [69,70]. Therefore, we propose that future research should focus on characterizing the specific functions of these three genes.

*L. formosana* leaves contain various high-value volatile compounds, including α-pinene, β-pinene, caryophyllene, and limonene. They are important raw ingredients in the pharmaceutical, chemical, and spice industries. At present, *L. formosana* leaf resources are underutilized because of a lack of good asexual lines, and the extraction of essential oils and chemical analysis methods are subject to the season, degree of growth and development, and other factors. We performed a fully integrated analysis of the transcriptome and volatile profiles in *L. formosana* leaves to gain fresh insight into how α-pinene and β-pinene accumulate and to distinguish the genes involved. These findings provide a promising pool of genes involved in the biosynthesis of α-pinene and β-pinene in *L. formosana* leaves. This offers a reference for further exploration into its economical, medicinal, and ecological values.

## 5. Conclusions

Overall, individual plants with significantly high (H) and low (L) contents of α-pinene and β-pinene were identified in this study. α-pinene and β-pinene were the most abundant volatile compounds in the “H group”.

Transcriptome sequencing data analysis showed that there were 1202 DEGs in group H vs. group L, including 689 upregulated and 513 downregulated genes. Specifically, DXR, HDS, and TPS may be the major regulators of leaf terpene biosynthesis. We identified three terpene synthases (*LfTPS3*, *LfTPS2, LfTPS1*) that were highly homologous to α-pinene and β-pinene synthases from other species in the phylogenetic analysis. The above three genes were identified as containing typical TPS domains, and four TPS genes (*LfTPS1*, *LfTPS2*, *LfTPS4*, and *LfTPS5*) were highly correlated with α-pinene and β-pinene. These may be critically involved in the biosynthesis and regulation of α-pinene and β-pinene according to interaction networks. Therefore, *LfTPS1* and *LfTPS2* are defined as the key candidate genes regulating the biosynthesis of α-pinene and β-pinene. This study sheds light on the genes involved in the biosynthesis of α-pinene and β-pinene in *L. formosana* leaves. The results lay the groundwork for future research on the functional characterization of candidate TPSs.

## Figures and Tables

**Table 1:** Transcriptome sequencing quality and mapping rate of leaves *L. formosana*.

Samples	Raw Reads	Clean Reads	GC Content (%)	Overall Mapping Rate
H1	15,540,839	7,080,812	49	86.91%
H2	7,256,585	6,559,084	49	86.54%
H3	17,427,878	15,922,369	49	86.89%
L1	17,887,504	16,267,056	49	88.13%
L2	11,780,757	10,731,248	48	87.31%
L3	13,997,784	12,768,237	49	88.50%

## Data Availability

All data supporting the findings of this study are available within the paper and its Supplementary Materials published online.

## Supplementary Materials

The following supporting information can be downloaded at: https://www.mdpi.com/article/10.3390/genes14010163/s1, Figure S1: Comparison of *L. formosana* leaf compounds in all samples.; Figure S2: shows ten predicted motifs; Table S1: Primer information used in qRT-PCR assays of five genes related to monoterpene pathways; Table S2: GC–MS detection data of the metabolome; Table S3: The expression of all the genes in the transcriptome.; Table S4: GO functional annotation and KEGG enrichment analysis data of DEGs.; Table S5: qRT-PCR results.; Table S6: Interaction network data between the 4 TPSs of *L. formosana*, α-pinene, and β-pinene.; Table S7: Provenance and accession number of terpene synthase.

## References

[1] Dezhu Li Y.S., Zhang S. (2020). Altingiaceae. Middle Volume of Genera of Vascular Plants in China.

[2] Zhang H.X., Kang Y., Li N., Wang H.F., Bao Y.R., Li Y.W., Li X.Z., Jiang Z., Chen G. (2020). Triterpenoids from *Liquidambar* Fructus Induced Cell Apoptosis via a PI3K-AKT Related Signal Pathway in SMMC7721 Cancer Cells. Phytochemistry.

[3] Xin S., XinHong L., DongYue J., YinGang L. (2019). Evaluation of Dust Retention and Antimicrobial Effect of 22 Common Landscape Plants Including *Liquidambar formosana*. J. Northeast For. Univ..

[4] Qin X., Wang L., Rao W. (2019). Study on the Antimicrobial Activity of Essential Oil from *Liquidambar fragrans*. China Pharm..

[5] Decarlo A., Zeng T., Dosoky N.S., Satyal P., Setzer W.N. (2020). The Essential Oil Composition and Antimicrobial Activity of *Liquidambar formosana* Oleoresin. Plants.

[6] Hu W.J., Wu Q., Liu X., Shen Z.J., Chen J., Liu T.W., Chen J., Zhu C.Q., Wu F.H., Chen L. (2015). Antioxidant Activity of Extract from *Liquidambar formosana* Hance Leaves and Its Effect on Preserving the Quality of Loquat Fruits During Refrigerated Storage. Food Sci..

[7] Ma H., Wang F., Jiang J., Cheng L., Zhang H., Zhang G. (2017). In Vivo Anti-Inflammatory Activity of *Liquidambar formosana* Hance Infructescence Extract. Trop. J. Pharm. Res..

[8] Liu Y., Liu Y., Liu Y., Liu H., Shang Y. (2018). Evaluating Effects of Ellagic Acid on the Quality of Kumquat Fruits During Storage. Sci. Hortic..

[9] Okmen G., Turkcan O., Ceylan O., Gork G. (2014). The Antimicrobial Activity of *Liquidambar orientalis* Mill. Against Food Pathogens and Antioxidant Capacity of Leaf Extracts. Afr. J. Tradit. Complement. Altern. Med..

[10] Zhong Y.T., Wang X.L., Xie Q.J., Zhang Y.N. (2016). Effect of the Extract from Leaves of *Liquidambar formosana* Hance on S180 Cells. Genet. Mol. Res..

[11] Yanjun S., Wei Z., Yigin W., Liao W., Wang L., Su W. (2019). Identification of Volatile Organic Compounds and Pharmaceutical Properties of Eight Common Urban Forest Plants from Shenzhen Regions. For. Environ. Sci..

[12] Chen X., Guo L., Xiao W., Chen X., Liu H., Guan Q. (2015). Study on Volatile Organic Compounds Released Characteristic of *Liquidambar formosana*. J. Southwest For. Univ. Nat. Sci..

[13] Zhenhai L. (2012). Study on Components and Emission Laws of BVOCs in Typical Forest Communities and by Their Main Trees in Purple Mountain in Nanjing. Ph.D. Thesis.

[14] Ling M., Jing Z., Ya-Hong Z., Bin W., Sai-Jun W., Jia-Xin L., Yan G. (2021). Seasonal Variation of Volatile Organic Compounds and Negative Air Ions in *Liquidambar formosana* Stand and Their Effect on Microbial Populations. Zhejiang For. Sci. Technol..

[15] Wang J., Chen Z., Han S., Lin F. (2015). Volatile Organic Compounds from Four Color-Leaf Tree Species. J. Zhejiang For. Sci. Technol..

[16] Chi-Jung C., Fang-Hua C., Shih-Chang C., Nai-Wen T., Sheng-Yang W. (2013). Comparative Analysis of Phytoncides Released from *Liquidambar formosana* Hance Trees and Seedlings. J. Agric. For..

[17] Nating X. (2013). Researches on Composition, Antimicrobial Activity and-Loquat’s Preservation of the Volatile Oil from Liquidambar formosana Hance Leaves. Ph.D. Thesis.

[18] Zhang Y.H., Feng J., Jin X.H., Yao X.Q., Yang Z.J. (2012). Components Analysis of Volatile Oil from *Liquidambar formosana* Leaves by GC-MS. Chin. J. Exp. Trad. Med. Formulae.

[19] Piechulla B., Bartelt R., Brosemann A., Effmert U., Bouwmeester H., Hippauf F., Brandt W. (2016). The **α**-Terpineol to 1, 8-Cineole Cyclization Reaction of Tobacco Terpene Synthases. Plant Physiol..

[20] Despinasse Y., Fiorucci S., Antonczak S., Moja S., Bony A., Nicolè F., Baudino S., Magnard J.L., Jullien F. (2017). Bornyl-Diphosphate Synthase from Lavandula angustifolia: A Major Monoterpene Synthase Involved in Essential Oil Quality. Phytochemistry.

[21] Radwan A., Kleinwächter M., Selmar D. (2017). Impact of Drought Stress on Specialised Metabolism: Biosynthesis and the Expression of Monoterpene Synthases in Sage (*Salvia officinalis*). Phytochemistry.

[22] Newman J.D., Chappell J. (1999). Isoprenoid Biosynthesis in Plants: Carbon Partitioning Within the Cytoplasmic Pathway. Crit. Rev. Biochem. Mol. Biol..

[23] Vranová E., Coman D., Gruissem W. (2013). Network Analysis of the MVA and MEP Pathways for Isoprenoid Synthesis. Annu. Rev. Plant Biol..

[24] Eisenreich W., Schwarz M., Cartayrade A., Arigoni D., Zenk M.H., Bacher A. (1998). The Deoxyxylulose Phosphate Pathway of Terpenoid Biosynthesis in Plants and Microorganisms. Chem. Biol..

[25] Lichtenthaler H.K. (1999). The 1-Deoxy-D-xylulose-5-Phosphate Pathway of Isoprenoid Biosynthesis in Plants. Annu. Rev. Plant Physiol. Plant Mol. Biol..

[26] Gupta A.K., Ganjewala D. (2015). A Study on Biosynthesis of “Citral” in Lemongrass (*C. flexuosus*) cv. Suvarna. Acta Physiol. Plant..

[27] Adam K.-P., Zapp J. (1998). Biosynthesis of the Isoprene Units of Chamomile Sesquiterpenes. Phytochemistry.

[28] Mccaskill D., Croteau R. (1995). Monoterpene and Sesquiterpene Biosynthesis in Glandular Trichomes of Peppermint (Mentha × piperita) Rely Exclusively on Plastid-Derived Isopentenyl Diphosphate. Planta.

[29] Adam K.P., Thiel R., Zapp J. (1999). Incorporation of 1-[1–13C] Deoxy-D-xylulose in Chamomile Sesquiterpenes. Arch. Biochem. Biophys..

[30] Towler M.J., Weathers P.J. (2007). Evidence of Artemisinin Production from IPP Stemming from Both the Mevalonate and the Nonmevalonate Pathways. Plant Cell Rep..

[31] Yu F., Utsumi R. (2009). Diversity, Regulation, and Genetic Manipulation of Plant Mono-And Sesquiterpenoid Biosynthesis. Cell. Mol. Life Sci..

[32] Ganjewala D., Luthra R. (2009). Geranyl Acetate Esterase Controls and Regulates the Level of Geraniol in Lemongrass (*Cymbopogon flexuosus* Nees ex Steud.) Mutant cv. GRL-1 Leaves. Z. Naturforsch. C J. Biosci..

[33] Rai A., Kamochi H., Suzuki H., Nakamura M., Takahashi H., Hatada T., Saito K., Yamazaki M. (2017). De Novo Transcriptome Assembly and Characterization of Nine Tissues of *Lonicera japonica* to Identify Potential Candidate Genes Involved in Chlorogenic Acid, Luteolosides, and Secoiridoid Biosynthesis Pathways. J. Nat. Med..

[34] Sallaud C., Rontein D., Onillon S., Jabès F., Duffé P., Giacalone C., Thoraval S., Escoffier C., Herbette G., Leonhardt N. (2009). A novel pathway for sesquiterpene biosynthesis from Z, Z-farnesyl pyrophosphate in the wild tomato *Solanum habrochaites*. J. Plant Cell.

[35] Matthews P.D., Wurtzel E.T. (2000). Metabolic engineering of carotenoid accumulation in Escherichia coli by modulation of the isoprenoid precursor pool with expression of deoxyxylulose phosphate synthase. J. Appl. Microbiol. Biotechnol..

[36] Peihuang Z., Yu C., Kongshu J. (2021). A Review of Terpene Synthases and Genes in Pinaceae. J. Nanjing For. Univ. Nat. Sci. Ed..

[37] Liu G.-F., Liu J.-J., He Z.-R., Wang F.-M., Yang H., Yan Y.-F., Gao M.-J., Gruber M.Y., Wan X.-C., Wei S. (2018). Implementation of CsLIS/NES in linalool biosynthesis involves transcript splicing regulation in *Camellia sinensi*. J. Plant Cell Environ..

[38] Bohlmann J., Meyer-Gauen G., Croteau R. (1998). Plant terpenoid synthases: Molecular biology and phylogenetic analysis. Proc. Natl. Acad. Sci. USA.

[39] Steele C.L., Crock J., Bohlmann J., Croteau R. (1998). Sesquiterpene synthases from grand fir (*Abies grandis*): Comparison of constitutive and wound-induced activities, and cDNA isolation, characterization, and bacterial expression of δ-selinene synthase and γ-humulene synthase. J. Biol. Chem..

[40] Chung D.D., Zhang W., Jatavallabhula K., Barrington A., Jung J., Aldave A.J. (2019). Alterations in GRHL2-OVOL2-ZEB1 Axis and Aberrant Activation of Wnt Signaling Lead to Altered Gene Transcription in Posterior Polymorphous Corneal Dystrophy. Exp. Eye Res..

[41] Yang Z., Xie C., Huang Y., An W., Liu S., Huang S., Zheng X. (2021). Metabolism and Transcriptome Profiling Provides Insight into the Genes and Transcription Factors Involved in Monoterpene Biosynthesis of Borneol Chemotype of *Cinnamomum camphora* Induced by Mechanical Damage. PeerJ.

[42] Yang Z., An W., Liu S., Huang Y., Xie C., Huang S., Zheng X. (2020). Mining of Candidate Genes Involved in the Biosynthesis of Dextrorotatory Borneol in *Cinnamomum burmannii* by Transcriptomic Analysis on Three Chemotypes. PeerJ.

[43] Kmieć K., Rubinowska K., Michałek W., Sytykiewicz H. (2018). The Effect of Galling Aphids Feeding on Photosynthesis Photochemistry of Elm Trees (Ulmus sp.). Photosynthetica.

[44] Tholl D. (2015). Biosynthesis and Biological Functions of Terpenoids in Plants. Biotechnology of Isoprenoids.

[45] Chen S., Zhang Y., Zhang T., Zhan D., Pang Z., Zhao J., Zhang J. (2022). Comparative Transcriptomic, Anatomical and Phytohormone Analyses Provide New Insights into Hormone-Mediated Tetraploid Dwarfing in Hybrid Sweetgum (*Liquidambar styraciflua* × *L. formosana*). Front. Plant Sci..

[46] Keeling C.I., Bohlmann J. (2006). Diterpene Resin Acids in Conifers. Phytochemistry.

[47] Vaughan M.M., Wang Q., Webster F.X., Kiemle D., Hong Y.J., Tantillo D.J., Coates R.M., Wray A.T., Askew W., O’Donnell C. (2013). Formation of the Unusual Semivolatile Diterpene Rhizathalene by the Arabidopsis Class I Terpene Synthase TPS08 in the Root Stele Is Involved in Defense Against Belowground Herbivory. Plant Cell.

[48] Yang C.Q., Wu X.M., Ruan J.X., Hu W.L., Mao Y.B., Chen X.Y., Wang L.J. (2013). Isolation and Characterization of Terpene Synthases in Cotton (*Gossypium hirsutum*). Phytochemistry.

[49] Kong D.X., Li Y.Q., Bai M., He H.J., Liang G.X., Wu H. (2017). Correlation Between the Dynamic Accumulation of the Main Effective Components and Their Associated Regulatory Enzyme Activities at Different Growth Stages in *Lonicera japonica* Thunb. Ind. Crops.

[50] Zhang L., Lu G., Huang X., Guo H., Su X., Han L., Zhang Y., Qi Z., Xiao Y., Cheng H. (2020). Overexpression of the Caryophyllene Synthase Gene GhTPS1 in Cotton Negatively Affects Multiple Pests While Attracting Parasitoids. Pest Manag. Sci..

[51] DeCarlo A., Johnson S., Okeke-Agulu K.I., Dosoky N.S., Wax S.J., Owolabi M.S., Setzer W.N. (2019). Compositional analysis of the essential oil of Boswellia dalzielii frankincense from West Africa reveals two major chemotypes. Phytochemistry.

[52] Pereira Da Silva H.N., Dos Santos Machado S.D., De Andrade Siqueira A.M., Cardoso Costa da Silva E., de Oliveira Canto M.Â., Jensen L., Vargas Flores da Silva L., Sena Fugimura M.M., de Sousa Barroso A., Veras Mourão R.H. (2020). Sedative and Anesthetic Potential of the Essential Oil and Hydrolate from the Fruit of *Protium Heptaphyllum* and Their Isolated Compounds in Colossoma Macropomum Juveniles. Aquaculture.

[53] Felipe C.F.B., Albuquerque A.M.S., De Pontes J.L.X., de Melo J.Í.V., Rodrigues T.C.M.L., de Sousa A.M.P., Monteiro Á.B., Ribeiro A.E.D.S., Lopes J.P., de Menezes I.R.A. (2019). Comparative Study of Alpha- And Beta-Pinene Effect on PTZ-Induced Convulsions in Mice. Fundam. Clin. Pharmacol..

[54] Salehi B., Upadhyay S., Erdogan Orhan I., Kumar Jugran A., Jayaweera S.L.D., Dias D.A., Sharopov F., Taheri Y., Martins N., Baghalpour N. (2019). Therapeutic Potential of Alpha- and Beta-Pinene: A Miracle Gift of Nature. Biomolecules.

[55] Nurzyńska-Wierdak R. (2009). Herb Yield and Chemical Composition of Common Oregano (*Origanum vulgare* L.) Essential Oil According to the Plant’s Developmental Stage. Herba Pol..

[56] Li Y., Zhou Y., Chen H., Chen C., Liu Z., Han C., Wu Q., Yu F. (2022). Transcriptomic Analyses Reveal Key Genes Involved in Pigment Biosynthesis Related to Leaf Color Change of *Liquidambar formosana* Hance. Molecules.

[57] Li J., Wang Y., Dong Y., Zhang W., Wang D., Bai H., Li K., Li H., Shi L. (2021). The Chromosome-Based Lavender Genome Provides New Insights into Lamiaceae Evolution and Terpenoid Biosynthesis. Hortic. Res..

[58] Kaur G., Arya S.K., Singh B., Singh S., Dhar Y.V., Verma P.C., Ganjewala D. (2019). Transcriptome Analysis of the Palma rosa Cymbopogon martinii Inflorescence with Emphasis on Genes Involved in Essential Oil Biosynthesis. Ind. Crops Prod..

[59] Nett R.S., Lau W., Sattely E.S. (2020). Discovery and Engineering of Colchicine Alkaloid Biosynthesis. Nature.

[60] Hafiz M.K. (2020). Identification, molecular cloning and Functional characterization of MEP Pathway genes (DXS, GPPS) from winterssweet flower. Ph.D. Thesis.

[61] Huang M., Fan R., Ye X., Lin R., Luo Y., Fang N., Zhong H., Chen S. (2018). The Transcriptome of Flower Development Provides Insight into Floral Scent Formation in *Freesia hybrida*. Plant Growth Regul..

[62] Nieuwenhuizen N.J., Chen X., Wang M.Y., Matich A.J., Perez R.L., Allan A.C., Green S.A., Atkinson R.G. (2015). Natural Variation in Monoterpene Synthesis in Kiwifruit: Transcriptional Regulation of Terpene Synthases by NAC and Ethylene-INSENSITIVE3-Like Transcription Factors. Plant Physiol..

[63] Mahmoud S.S., Croteau R.B. (2001). Metabolic Engineering of Essential Oil Yield and Composition in Mint by Altering Expression of Deoxyxylulose Phosphate Reductoisomerase and Menthofuran Synthase. Proc. Natl. Acad. Sci. USA.

[64] Ma Z.Z., Pang X.Q., Sheng R., Pei X.L., Wang Q.Y., Xie T., Yin X.P. (2015). Research Advances of Key Enzymes in the Biosynthesis Pathways of Isoprenoids. J. Hangzhou Norm. Univ. Nat. Sci. Ed..

[65] Fengying Q. (2020). Study on Chemical Composition of Leaf Essential Oil and the Genes Related to the Biosynthesis of Important Terpenoids in *Cinnamomum porrectum*. Ph.D. Thesis.

[66] Mancarz G.F.F., Laba L.C., da Silva E.C.P., Prado M.R.M., de Souza L.M., de Souza D., Nakashima T., Mello R.G. (2019). *Liquidambar styraciflua* L.: A new potential source for therapeutic uses. J. Pharm. Biomed. Anal..

[67] Landmann C., Fink B., Festner M., Dregus M., Engel K.H., Schwab W. (2007). Cloning and Functional Characterization of Three Terpene Synthases from Lavender (*Lavandula angustifolia*). Arch. Biochem. Biophys..

[68] Külheim C., Padovan A., Hefer C., Krause S.T., Köllner T.G., Myburg A.A., Degenhardt J., Foley W.J. (2015). The Eucalyptus Terpene Synthase Gene Family. BMC Genom..

[69] Rajaonarivony J.I., Gershenzon J., Croteau R. (1992). Characterization and Mechanism of (4S)-Limonene Synthase, a Monoterpene Cyclase from the Glandular Trichomes of Peppermint (Mentha x piperita). Arch. Biochem. Biophys..

[70] Colby S.M., Alonso W.R., Katahira E.J., McGarvey D.J., Croteau R. (1993). 4S-Limonene Synthase from the Oil Glands of Spearmint (*Mentha spicata*). cDNA Isolation, Characterization, and Bacterial Expression of the Catalytically Active Monoterpene Cyclase. J. Biol. Chem..

