# Supplementary figures and images for: Volatile Profiling and Transcriptome Sequencing Provide Insights into the Biosynthesis of α-Pinene and β-Pinene in Liquidambar formosana Hance Leaves
Source: Genes (Basel). 2023 Jan 6;14(1):163. doi: 10.3390/genes14010163 (PMC9858688; doi:10.3390/genes14010163)

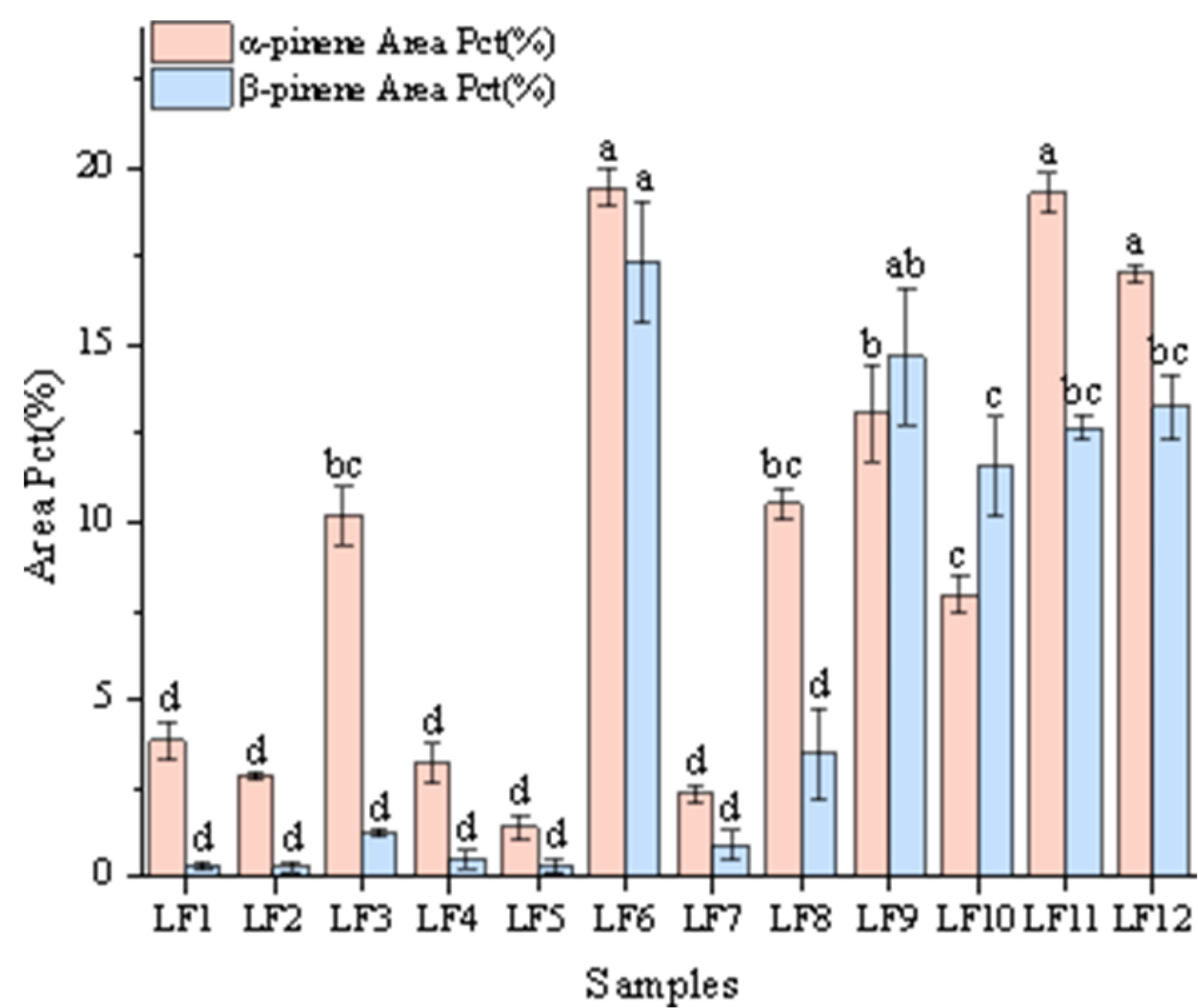

Supplement: Supplementary file 1 [file genes-14-00163-s001.zip › Supplementary Figure S1. Comparison of compounds in L. formosana leaf of all samples..pdf]

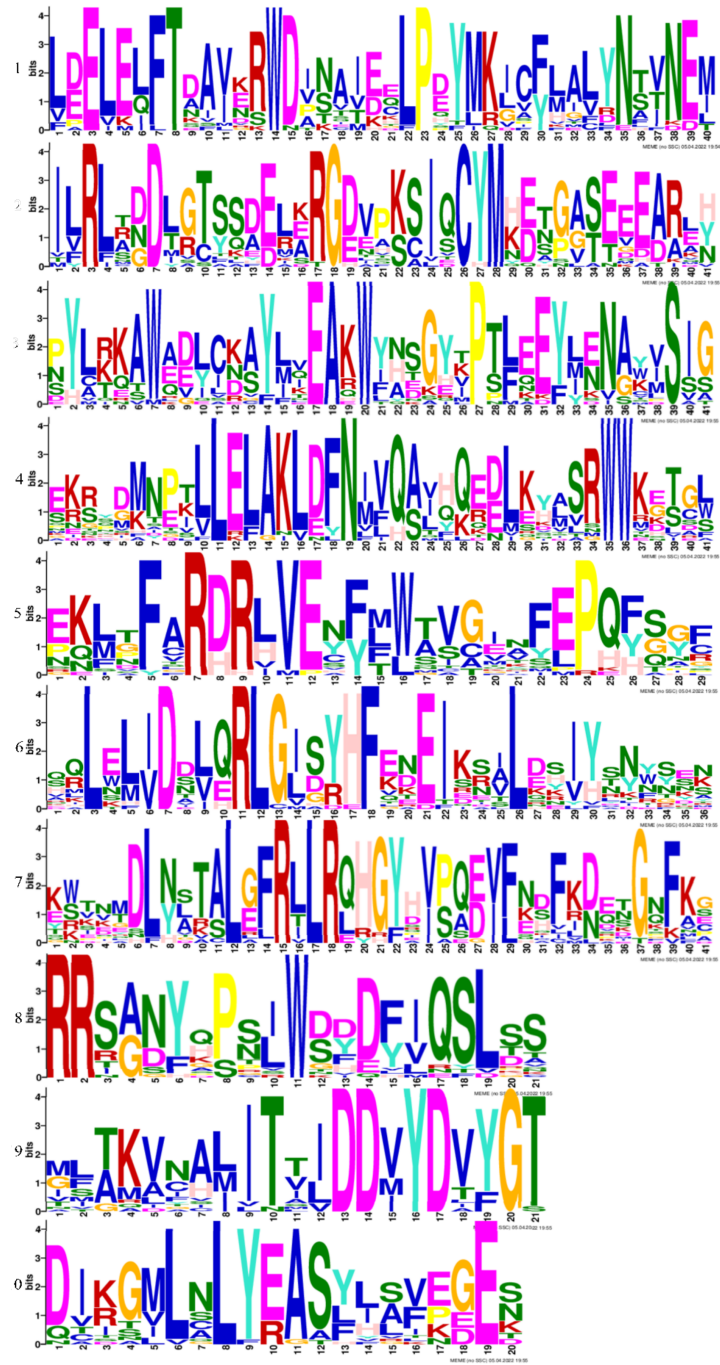

Supplement: Supplementary file 1 [file genes-14-00163-s001.zip › Supplementary Figure S2. shows 10 predicted motif..pdf]
